# The effect of ABM on attentional networks and stress-induced emotional reactivity in a mixed clinical sample with depression: A randomized sham-controlled trial

**DOI:** 10.1016/j.nsa.2024.104091

**Published:** 2024-09-24

**Authors:** Ragnhild Bø, Brage Kraft, Rune Jonassen, Jutta Joormann, Catherine J. Harmer, Nils Inge Landrø

**Affiliations:** a Clinical Neuroscience Research Group, University of Oslo, Oslo, Norway; b Division of Psychiatry, Diakonhjemmet Hospital, Norway; c Faculty of Health Sciences, Oslo Metropolitan University, Norway; d Affect Regulation and Cognition lab, Yale University, USA; e Department of Psychiatry, Oxford University, United Kingdom; f Oxford Health NHS Foundation Trust, Warneford Hospital, Oxford, OX3 7JX, UK, United Kingdom

**Keywords:** Attention bias modification, Attentional networks, Stress, Reactivity, Depression

## Abstract

Research on the efficacy of Attention Bias Modification for depressive symptoms has predominantly yielded unfavorable outcomes. Despite adhering to rigorous conventions in randomized controlled trials, findings from these studies have indicated minimal effect sizes, thereby raising concerns about their limited clinical significance. This may be attributed to the overlapping mechanisms in ABM and the sham comparator, both affecting attentional processes. Participants with a diagnosis of major depressive disorder, with and without comorbid anxiety (N = 101) were randomized to a two-week preregistered randomized trial of ABM compared to sham. Attentional networks were assessed prior to and after the intervention by the Attention Network Task (ANT), and emotional reactivity was assessed in response to a lab-stressor. Irrespective of condition, participants improved their performance on the alerting and executive attentional networks, but not orienting, and stress-induced emotional reactivity was marginally decreased. Changes in attentional networks predicted post-intervention depression scores. It is imperative to reconsider the employment of a sham comparator in the exploration of the clinical efficacy of ABM.

## Highlights

• Attention bias modification (ABM) has yielded mostly null findings for depression.
• Due to its close resemblance, the sham condition may hide some of the effect of ABM.
• Both conditions improved alerting and executive attentional networks.
• Improvements predicted level of depression post-intervention.
• The use of sham control must be reevaluated for efficacy trials.

## 1 Background

Emotion and attention are intricately linked, with emotions often influencing the allocation of attention, and attention in turn shaping emotional experiences (Yiend, 2010). For example, individuals with heightened attentional biases toward negative or threat-related stimuli are more susceptible to experiencing negative emotional reactions (Fox et al., 2010), and may be at an elevated risk for the development and perpetuation of mood disorders. This suggests a potential link between attentional processes, emotional reactivity, and the susceptibility to mood-related disorders (Disner et al., 2017). Accordingly, attention biases (AB), are found in all phases of a depressive disorder (Shamai-Leshem et al., 2022). Attention bias modification (ABM) aims at modify these biases through implicitly tuning attention towards relatively more positive stimuli by means of positive reinforcement (Browning et al., 2012), thereby reducing emotional reactivity to stressors (Amir et al., 2008) with the ultimate goal of improving mental health outcomes.

The dot probe paradigm is frequently employed for modifying AB (MacLeod and Mathews, 2012). During training, by introducing a contingency between the probe and positive stimuli (e.g., facial expressions), the participant is implicitly taught where to allocate attention to effectively conduct the task. Traditionally, a sham procedure without a specific contingency between the probe and the stimuli, has been employed as the control condition. Hence, the only difference between the active ABM and the sham, is the allocation of the probe relative to the valence of the emotional stimuli, supposedly distilling AB modification as the active mechanism of the intervention.

AB is assumed to originate from the increased exertion from subcortical emotion processing regions not being effectively succumbed by top-down cognitive control (Disner et al., 2011; Rock et al., 2014). At a neuronal level, ABM compared to sham has been associated with reduced activation in amygdala, midline structures and ACC in response to negative stimuli, and also associated with stronger connections within the salience networks (Hilland et al., 2018, 2020). Hence, ABM has been found to modify both the functional and structural basis of emotion generation and appraisal.

Theory and empirical findings, however, do not always go hand in hand, and the overall effect sizes attributable to the ABM intervention for depressive symptoms are small (Fodor et al., 2020), and according to a recent meta-analysis the current evidence base is inconclusive (Xia et al., 2023). It has been speculated that the failure to detect the superiority of ABM over sham in reducing symptoms could be attributed to the lack of effect on the intended mechanism (i.e., reduction in negative AB (Grafton et al., 2017). However, the lack of effect on symptoms when ABM is compared to the sham procedure could also be explained by close resemblance between the procedures (Blackwell et al., 2017). Both ABM and sham require the patient to watchfully observe emotional stimuli and respond quickly to indicate where a probe appears. The only difference between the procedures is the contingency of the probe. Thus, there is a possibility that both conditions may lead to improvements in attentional function (e.g., improvement in alerting and executive networks among anxious samples (Heeren et al., 2015; McNally et al., 2013)), though not found based on self-report among depressed samples (unpublished master theses referred to in meta-analysis by Xia, Li (Xia et al., 2023)).The relative difference between the conditions on symptom reductions may therefore be small.

Attentional processes can be described as selective focus on salient environmental features while ignoring other aspects. Hence, they play a pivotal role in various cognitive functions, such as working memory, decision-making, and emotion regulation. Based on function and neuroanatomy, the attentional system can be mapped on to three separate components, namely alerting, orienting and executive (Fan et al., 2005). Attentional processes (i.e., orienting) are associated with the volatility of emotional reactions (i.e., emotional reactivity) in response to stressors (Bø et al., 2023a). While high emotional reactivity in response to stress typically is considered negative (Schlotz and Gellman, 2020) and also is associated with the severity of depressive symptoms (Bø et al., 2023a), blunted emotional reactivity (Bylsma et al., 2008) and attentional impairment (Keller et al., 2019) has also been found to be a hallmark of depression.

Our group has investigated the effect of ABM on depression symptoms in a randomized controlled-trial (RCT) of 101 participants with depression and comorbid disorders (Bø et al., 2023b). The RCT evaluated the efficacy based on a comparison between ABM and a sham condition, with results showing no difference in symptom change between the conditions. However, both conditions lead to symptom reduction during the intervention period, and thereby replicated the findings of several other similar studies (Basanovic et al., 2020; Carleton et al., 2016; De et al., 2017). This pattern was also consistent with the findings from the largest ABM study conducted on depression to date, wherein improvements across conditions were notably more substantial than the distinctions observed between ABM and sham interventions (Jonassen et al., 2019). In the present study, we examined whether ABM or sham is followed by changes in attentional processes and how these changes are related to stress-induced emotional reactivity post intervention and symptom improvement. We hypothesized that both conditions, ABM and sham, result in improved attentional function from pre-to-post intervention. Following previous studies demonstrating an effect on attentional networks following ABM for anxiety (Heeren et al., 2015; McNally et al., 2013), we expected that performance in alerting and executive networks will improve and be associated with decreased stress-induced emotional reactivity.

## 2 Materials and methods

### 2.1 Participants

The present study includes participants from the trial #NCT04137367 (clinicaltrials.gov) reported in Bø, Kraft (Bø et al., 2023b) that underwent the ABM vs. sham intervention and includes the pre-and post-intervention assessments of attentional networks and stress-induced emotional reactivity.

Participants (N = 101) were recruited through local advertisements and in social media. All participants were assessed using the MINI International Neuropsychiatric Interview PLUS 5.0.0 (M.I.N.I.). For inclusion, the participants needed to be between the ages 18–65, fulfill the criteria for MDD, with or without an anxiety disorder and with or without alcohol use disorder. Exclusion criteria were mania, psychosis, and neurological disorder, but not hypomania, thus allowing participants with Bipolar-II to enter the trial. See Table 1 for details of the sample.

**Table 1:** Participant characteristics.

	Sham (*n* = 51)	ABM (*n* = 50)	*p*
Sex (female)	41 (80%)	32 (64%)	.066
Age (years)^a^	44 (11.3)	44 (10.3)	.789
Education level (ISCED)^a^	5.7 (1.3)	5.6 (1.3)	.785
Ongoing MDD	20 (39%)	27 (54%)	.139
Previous MDD	48 (94%)	48 (96%)	.667
MDD in remission	31 (61%)	23 (46%)	.139
Current antidepressant medication	14 (27 %)	20 (40%)	.099
Current comorbid anxiety disorder	37 (73%)	34 (68%)	.780
BDI-II^a^	26.3 (9.8)	23.1 (10.2)	.149

*Note.* Data are *n* (%) or *mean* (*SD*).

ABM = Attentional Bias Modification, BDI-II = Beck's Depression Inventory-II, ISCED = International Standard Classification of Education, MDD = Major depressive disorder.

a Data not available for all randomized participants. Please see text for details.

### 2.2 Intervention

**Fig. 1:**
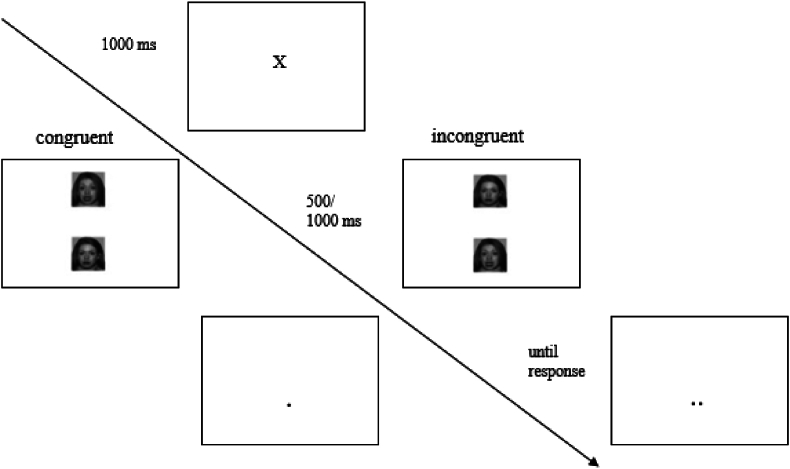
Attention Bias Modification *Note.* In the ABM condition, 87 % of the trials were congruent (dots presented in the location of the more positive stimuli), whereas in the sham condition there was no contingency (50/50 %).

The computerized ABM intervention was based on a dot-probe task, where vertically paired pictures displaying negative (i.e., anger, fearful) positive (i.e., happy) and neutral facial expression were presented on a laptop screen (Browning et al., 2012; Jonassen et al., 2019). The three pairs were randomly displayed on the screen for either 500 or 1000 ms in a total of 96 trials on a 1:1:1-ratio. After stimulus presentation, one or two dots appeared in the location of either one of the pictures, requiring the participants to indicate the number of dots quickly and accurately by keyboard presses. In the active condition, the dots were presented in the location of the relatively more positive stimuli in 87 % of the trials, whereas in the sham condition the dots had no contingency between probe location and the valence of the stimuli. The contingency was unknown to the participants. The intervention consisted of 28 sessions, performed twice daily for 14 days at laptop computers provided from the research group for this purpose only, resulting in 2688 trials in total. See Fig. 1 for illustration.

### 2.3 Measures

#### 2.3.1 Beck's Depression Inventory-II (BDI-II)

We used the BDI-II (Beck et al., 1988) for investigating self-reported depressive symptoms. The inventory consists of 21 items, and participants responds on 4-point Likert scale (0–3). Cronbach's alpha was: *α* = .870 at baseline and *α* = .970 post intervention.

#### 2.3.2 Attention Network Task (ANT)

**Fig. 2:**
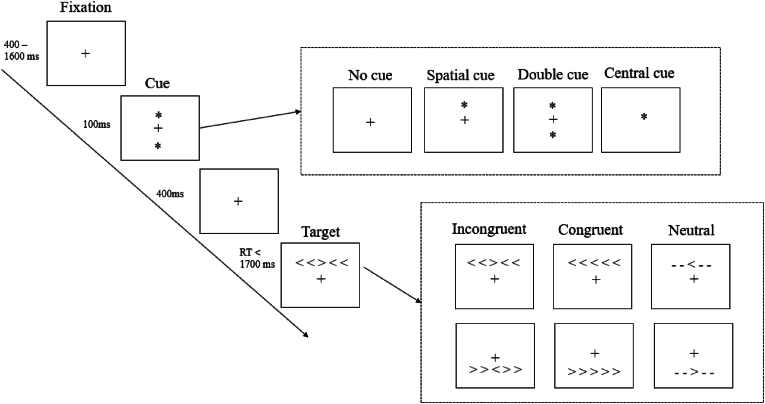
Attention Network Test *Note.* Schematic illustration of the Attention Network Test (Fan et al., 2002).

ANT (Fan et al., 2002) assesses three separate attentional networks; «alerting», «orienting» and «executive». In this computerized task, the participants are instructed to press buttons corresponding to right- or left-pointing arrows shown on a screen. They are asked to be as fast and accurate as possible, and reaction times are recorded for each trial. The ANT consists of a practice block and three experimental blocks of 96 trials. Each trial starts with a fixation cross, thereafter a cue (no cue, center cue, double cue, or spatial cue), a new fixation cross, and then the target (central arrow pointing left or right), flanked by congruent or incongruent arrows or neutral stimuli. See Fig. 2 for illustration.

Performance of the alerting network is assessed by calculating the difference in reaction time when a warning signal is present versus not present (i.e., double cue vs no cue), and with lower values reflecting poorer performance. The orienting network is assessed by differences in reaction time when a cue reflects spatial location of the target versus not (i.e., spatial cue vs. central cue), and lower values are indicative of lower performance. Performance of the executive network is assessed by the difference in reaction time when the central arrow is surrounded by congruent versus incongruent flankers, with higher scores reflecting poorer functioning (DeJong et al., 2019). The test-retest correlation coefficient for the ANT in clinical and non-clinical populations is moderate for executive, (*r* > .5 in HC and psychiatric populations), moderate to weak for orienting (*r* > .34 - .53) and weak for alerting (*r* > .23-.32) (Hahn et al., 2011).

#### 2.3.3 Stress-induced emotional reactivity

Emotional reactivity was assessed using a stress-induction paradigm in a laboratory. Stress induction was conducted according to the imagery scripts approach developed by Sinha and Tuit (2012). Details of the procedure is described in (Bø et al., 2023a). In short, participants were interviewed about a recently experienced stressful event that had made them either “sad, mad, or upset”. In addition, they responded to a checklist of the associated bodily sensations they felt in that situation. This allowed us to create personalized scripts, recorded in the voice of the researcher, that included autobiographical accounts of a recent stressful situation. The participants listened to the script by wearing headphones and they were asked to keep their eyes closed, if possible. This was done to facilitate visual mental imagery of the event.

The effects of stress induction were measured by changes in self-reported stress and mood from pre to post induction. We employed a visual analogue scale (VAS) in measuring subjective stress, asking participants to indicate how stressed they felt in the moment on a scale from 0 to 100 (0 = not at all, 100 = extremely stressed). The shortened version of the Profile of Mood States (POMS; Shacham, 1983) includes 37-items, is scored on a 5-point Likert scale (0–4), and has six sub-scales: depression, anxiety, fatigue, activity, confusion, and anger. The average score on POMS depression (Cronbach's *α*: pre induction = .946, post induction = .934) was used as an indicator of depressive mood. Stress-induced emotional reactivity was operationalized as the difference in depressed mood from pre-to post-induction as measured by POMS. Higher scores reflect greater reactivity (i.e., increases in depressive mood from pre-to post-induction).

### 2.4 Procedure

**Fig. 3:**
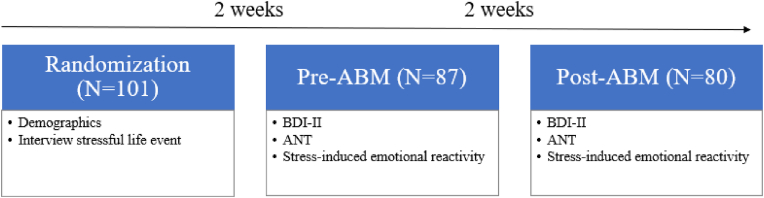
Timeline of the study *Note.* ABM = attentional bias modification, ANT = attention network task, BDI-II = Beck's depression inventory.

Participants were randomized to either ABM or sham. Before the intervention took place, they performed ANT and underwent the stress induction procedure and filled out momentary measures of stress and mood prior to and after listening to the script. After 14 days of ABM, performed twice daily at home on laptop computers provided by the research team, the participants returned to the lab performed ANT a second time and underwent a repeated stress induction and the associated assessments of stress and mood. The same scripts were used for both stress induction procedures. See Fig. 3 for timeline of the study. The parent trial was preregistered with clinicaltrials.gov (#NCT 04137367).

### 2.5 Statistical analysis

All data were analyzed in SPSS 27.0 (IBM) and STATA 17.0. Baseline characteristics of participants in the two intervention groups were reported using frequency distributions and descriptive statistics including measures of central tendency and dispersion. We conducted four separate mixed model analyses with random intercepts and unstructured covariance matrix investigating the effect of ABM on attentional networks (alerting, orienting and executive) and stress-induced emotional reactivity using an intention-to-treat approach and included all randomized participants who had either pre-or post-intervention data. The model was fit using restricted maximum likelihood in STATA, and ABM effect was operationalized as the least square mean difference at follow-ups from these mixed-model repeated measure models. By means of a multiple regression model, we investigated whether changes in attention and stress-reactivity predicted post-intervention depression scores. To calculate change, pre-intervention scores were subtracted from post-interventions scores. Hence, negative values for BDI-II, stress-induced emotional reactivity and ANT executive implies improvement (i.e., reductions in symptoms, reactivity, and interference effect), whereas positive values for ANT alerting and ANT orienting implies improvement (i.e., improved attentional performance and bias away from negative stimuli). To investigate whether sample heterogeneity predicted change, exploratory multiple regression analyses was conducted. Variables were inspected for outliers. Statistical significance was set at *p* = .05. Marginally significant results are also reported.

#### 2.5.1 Missing data

One-hundred-and-one participants were randomized, and 87 met for baseline assessment. Eighty participants completed the intervention: 42 in the sham condition and 38 in the ABM condition. Due to technical problems, reactivity data is missing for six participant pre-intervention and two participants at post-intervention. ANT data was available for 82 participants pre intervention and for 71 participants post-intervention.

### 2.6 Ethical considerations

The study was conducted in accordance with the Helsinki Declaration and the ethical principles for Nordic Psychologists, as issued by the Norwegian Psychological Association. The Regional committees for medical and health research ethics (2019/330) approved the study. All subjects provided written informed consent before the study was undertaken.

## 3 Results

### 3.1 Participants

On average, this was a sample with high educational level, where the majority is women having depressive symptoms in the moderate range and a comorbid anxiety disorder. A minority were currently on psychopharmacological medication. Also see (Bø et al., 2023b) for full details.

### 3.2 Manipulation check

We refer to Bø, Kraft (Bø et al., 2023a) for information on the effect of the stress-induction on self-reported stress and mood pre-intervention. See supplementary for details regarding post-intervention. In summary, the stress-induction had the expected effect of increasing we stress and emotional reactivity in the majority of the participants both pre-and post-intervention.

### 3.3 The effect of ABM on attentional networks

#### 3.3.1 Alerting

**Fig. 4:**
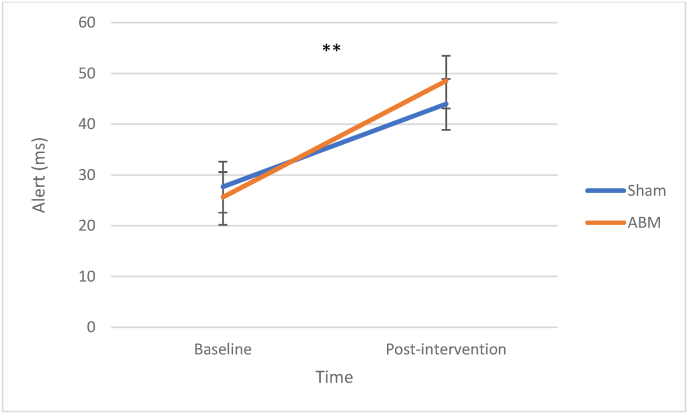
Alerting Attention at Baseline and After Two Weeks of ABM *Note.* Increasing reaction times indicate better function. ABM = attention bias modification. ∗∗*p* < .01.

There was a significant improvement in alertness from baseline to post-intervention, *β* = 16.37, SE = 6.26, *z* = 2.62, *p* = .009. There was no main effect of ABM condition, *β* = −1.99, SE = 6.87, *z* = −.28, *p* = −.776. There was no significant interaction effect between ABM and assessment point from baseline to post-intervention, *β* = 6.55, SE = 9.03, *z* = .72, *p* = .469. See Fig. 4 for illustration.

#### 3.3.2 Orienting

There was no significant change in orienting from baseline to post-treatment, *β* = −4.00, SE = 7.17, *z* = −.56, *p* = .577. There was no main effect of ABM condition, *β* = 6.59, SE = 9.3, *z* = .71, *p* = .478. There was no significant interaction effect between ABM and assessment point from baseline to post-treatment, *β* = −1.04, SE = 10.36, *z* = −.10, *p* = .920.

#### 3.3.3 Executive

**Fig. 5:**
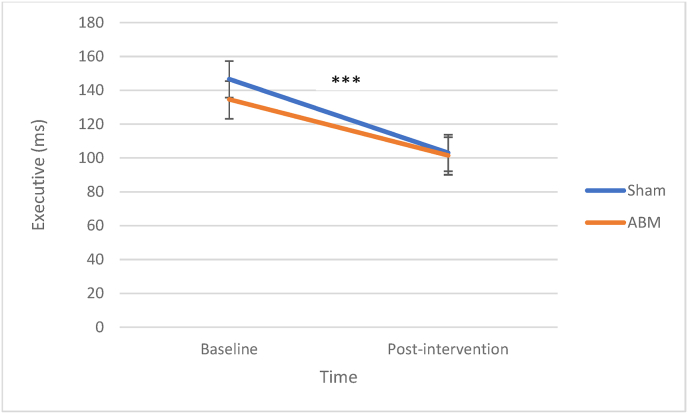
Executive Attention at Baseline and After Two Weeks of ABM *Note.* Decreasing reaction times indicate better performance. ABM = attention bias modification. ∗∗∗*p* < .001.

Visual inspection of the data showed that two subjects had extreme scores (>4 SD) post-intervention (one from the ABM group and one from the sham group), and these were also showing extreme changes (>4 SD) from pre-to-post intervention. These observations were excluded as statistical outliers. There was a significant improvement in executive from baseline to post-treatment, *β* = −43.59, SE = 7.87, *z* = −5.54, *p* < .001. There was no main effect of ABM condition, *β* = −12.08, SE = 15.43, *z* = −.80, *p* = .43. There was no significant interaction effect between ABM and assessment point from baseline to post-treatment, *β* = 10.47, SE = 11.24, *z* = .93, *p* = .359. Inclusion of outliers rendered the results of the MLM-model unchanged. See Fig. 5 for illustration.

### 3.4 The effect of ABM on stress-induced emotional reactivity

**Fig. 6:**
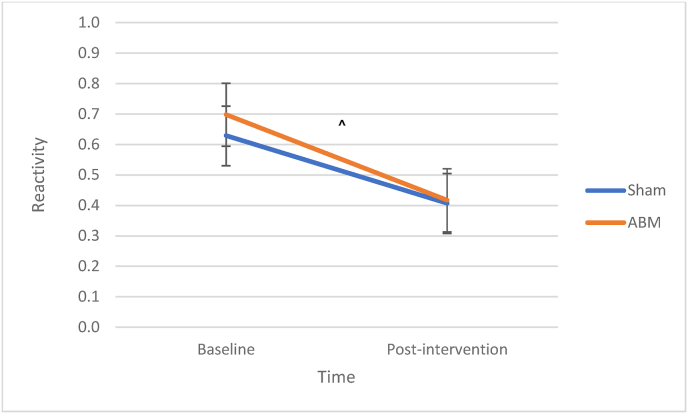
Stress-Induced Emotional Reactivity at Baseline and After Two Weeks of ABM *Note.* Lower values indicate less reactivity. ABM = attention bias modification. ^ *p* < .06.

There was a marginally significant decrease in stress-induced emotional reactivity from baseline to post-intervention, *β* = −.22, SE = .12, *z* = −1.81, *p* = .070. There was no main effect of ABM condition, *β* = .07, SE = .18, *z* = −.32, *p* = .63. There was no significant interaction effect between ABM and assessment point from baseline to post-intervention, *β* = −.06, SE = .18, *z* = −.32, *p* = .75. See Fig. 6 for illustration.

### 3.5 Changes in ANT and stress-reactivity predict depression

We conducted a multiple regression analysis with the variables that changed significantly during the intervention as predictors (change scores for ANT attention, ANT executive, and stress-reactivity), and depression as the outcome. The overall model (Table 2) was statistically significant, adj. R^2^ = .087, F (3, 56) = 2.877, *p* = .044. Due to the small sample size, bootstrapping with 5000 samples was conducted to ensure robustness of the estimates. Bootstrapped 95 % confidence intervals showed that improvements in ANT alerting and ANT executive predicted lower depression scores.

**Table 2:** Multiple regression analysis predicting post-intervention depression scores.

	b	SE	β	t	*p*	Bootstrap 95% CI	
						LB	UP
Intercept	20.058	1.190		10.558	<.001	16.636	24.219
Change in ANT alerting (n = 68)	−.072	.036	−.250	−1.987	.034	−.137	−.008
Change in ANT executive (n = 66^a^)	.057	.031	.239	1.811	.094	.002	.130
Change in reactivity (n = 73)	−2.484	1.942	−.170	−1.279	.177	−6.252	1.180

*Note.* ANT = Attention Network Test. b = beta, CI = confidence interval, LB = lower bound, SE = standard error, UP = upper bound.

a Bootstrap confidence interval [-.006, .085] when including two outliers (>4SD above mean).

### 3.6 The effect of clinical characteristics

To investigate the role of heterogeneity in our sample, we conducted multiple regression analyses to investigate if the use of antidepressants, ongoing MDD, and ongoing comorbid anxiety disorder predicted changes in the ANT alerting, ANT executive, or reactivity. Regarding ANT alerting, results showed that the model was non-significant F (3, 63) = 2.074, *p* = .113, with comorbid anxiety disorder as the only significant predictor, t = 2.154, *p* = . 035. Hence, comorbid anxiety disorder was associated with larger changes in ANT alerting. Regarding ANT executive and reactivity, the models were not significant (*p*'s > .58), and neither were any of the predictors (all *p*'s > .23).

## 4 Discussion

This study investigated the effect of ABM compared to sham on an objective measure of attention and on self-reported stress-induced emotional reactivity in a mixed clinical sample. Across conditions, we found improvements from pre-to-post intervention in attentional networks, specifically alertness and executive, and marginal improvements in stress-induced emotional reactivity. There was no difference between conditions on any of the outcomes. This pattern of results corroborates the main finding from the parent trial (Bø et al., 2023b), in which no differences between ABM and sham conditions were detected on either symptom severity, attentional bias, nor rumination. However, reduced symptom severity and reduced rumination was found after the intervention across conditions.

### 4.1 Attention

In our mixed clinical sample, we observed improved performance in alerting and executive attentional networks after the intervention period. Hence, our findings corroborate the results by (Heeren et al., 2015; McNally et al., 2013), who also found improvements in the same networks in anxious samples after two and four sessions of ABM, respectively. However, since we lack a control group representing natural trajectories and the effect of repeated testing in attentional performance, we cannot discern whether our findings are due to the intervention, practice effects, or the passage of time.

The improvement in attentional networks throughout the intervention period was specific to alerting and executive attentional networks. Curiously, attention training has been found to be more effective when targeting orienting networks (Peng and Miller, 2016), and attentional biases for negative material may originate from deficient orienting networks (e.g. Moriya and Tanno, 2009). Orienting may also be implicated in distress proneness and stress-induced emotional reactivity (Bø et al., 2023a; Rothbart et al., 1994). This might suggest that intervention that more specifically target the orienting network perhaps could constitute a future direction for reducing vulnerability for mood-related disorders, perhaps by impacting AB.

From pre-to-post intervention, depression levels in both conditions were reduced (Bø et al., 2023b), and the improvements in executive attention and alerting attention, contributed significantly to post-intervention depression scores. In anxious samples, this association has not been found (Heeren et al., 2015; McNally et al., 2013) and suggests that the association between improvements of attentional networks and symptoms are complex, and perhaps more prominent in depression compared to anxiety. Still, our exploratory analysis suggested that the presence of comorbid anxiety disorder might be of relevance for improvements in the alerting component of attention. More studies investigation how modification of attentional networks affects depression and the specific symptoms of depression, are required.

### 4.2 Stress-induced emotional reactivity

Previously, ABM has been found to reduce emotional reactivity to stressors (Amir et al., 2008). This effect was not evident in our data when contrasting ABM to a sham-controlled condition, and stress-induced emotional reactivity was only marginally reduced from pre-to post-intervention. Also, change in reactivity did not predict post-intervention depression scores. This could be attributed to the lack of significant change in AB in the ABM condition (e.g., Julian et al., 2012), but it could also be indicative of the limitations with self-reports, particularly considering demand characteristics in experimental setups. The repeated utilization of the same stress-induction scenarios may have introduced a habituation to the procedure and reduced the sensitivity of the reactivity measure at post intervention. Obtaining objective data on stress-induced emotional reactivity (i.e., depression), for example in terms of passively obtained behavioral data (De Angel et al., 2022), or biological markers of stress activation (e.g., cortisol), could be important in future trials.

### 4.3 Internal validity

The reliability when assessing the attentional networks by means of ANT has been questioned (e.g., Ishigami and Klein, 2010; Wang et al., 2014). Moreover, there are known reliability issues with difference scores, in particular in cases where variables are strongly correlated (McNally, 2019). In the present study, the correlations between time points were in the small to moderate range (*r* = .223-.477). The difference scores may be even less reliable due to doubling of the error terms. Hence, ensuring that variables can be reliably assessed will be important for the validity of future studies investigating change.

Also, the mere passing of time and practice effects associated with repeated testing may have given rise to the findings. An assessment only condition would have brought clarity to whether repeated exposures or the intervention were the main source of these findings.

While this trial was preregistered, these specific analyses were not, and must therefore be considered exploratory. The study may have been underpowered to demonstrate an ABM × Time interaction effect, and larger scale trials are needed to reduce the risk for Type II-errors. On the other hand, if the findings are so small that they may only be demonstrated in larger samples, the clinical significance of the findings may be questionable.

### 4.4 External validity

Our sample consisted of participants in different phases of their depressive disorders, some had comorbid anxiety disorders, and some were on psychotropic medications. While this mix is typical for a general psychiatric population, it also adds noise to the results and subsequently the conclusions that can be drawn, and this represents a limitation in the present study. On the overall, however, analyses suggested that these characteristics were not especially relevant in explaining changes in attention and reactivity. A study including a more homogenous sample would have avoided this problem and made the interpretation of the results more straightforward, but at the cost of generalizability.

### 4.5 Implications for future trials

The ABM-intervention and the sham condition differ only based on the contingency of targets, implying that some common mechanism might impede the identification of ABM's effectiveness on symptom level changes in clinical populations. In past years, large-scale clinical trials have yielded mostly null findings when investigating ABM for depressive symptoms (Bø et al., 2023b; Basanovic et al., 2020; de Voogd et al., 2016), but see (Browning et al., 2012; Yang et al., 2015, 2016) for positive findings among smaller-scale studies). It has been proposed that the clinical effectiveness of ABM cannot be demonstrated when the intervention is compared to sham (Blackwell et al., 2017), potentially due to some common effect in both conditions. Hence, future trials for demonstrating clinical efficacy may need to adopt new control conditions (Blackwell et al., 2017; Mogg et al., 2017). Landrø, Harmer (Landrø et al., 2023), for example, discusses an alternative active control procedure including repeated assessments of mood and bias, but no sham procedure.

## 5 Conclusion

Compared to the control condition, there was no effect of ABM on attentional networks and stress-reactivity. However, improvements in alerting and executive attentional networks were observed across both conditions, and these improvements predicted post-intervention depression scores. We advocate for the need of employing control conditions other than sham when evaluating the effectiveness of this type of computerized interventions in clinical populations.

## Funding

Foundation Dam (2019/FO249225), South-Eastern Norway Regional Health Authority (2020021), University of Oslo, and NIHR Oxford Health Biomedical Research Centre. The funders of the study had no role in study design, data collection, data analysis, data interpretation, or writing of the report.

## CRediT-statement

Conceptualization: Ragnhild Bø; Methodology: Ragnhild Bø;, Nils Inge Landrø;, Catherine J. Harmer, Rune Jonassen, Jutta Joormann; Formal analysis and investigation: Ragnhild Bø;, Brage Kraft; Writing - original draft preparation: Ragnhild Bø; Writing - review and editing: all authors; Funding acquisition: Nils Inge Landrø; Supervision: Nils Inge Landrø.

## Declaration of competing interest

CJH has received consultancy fees from P1vital, Lundbeck, Sage Therapeutics, Compass Pathways, Zogenix outside of this work. Other authors have no conflicts to declare.
