# Supplementary material for: The effect of ABM on attentional networks and stress-induced emotional reactivity in a mixed clinical sample with depression: A randomized sham-controlled trial
Source: Neurosci Appl. 2024 Sep 24;3:104091. doi: 10.1016/j.nsa.2024.104091 (PMC12244014; doi:10.1016/j.nsa.2024.104091)
Supplement: Multimedia component 1 [file mmc1.docx]

**Supplementary Materials**

Post-intervention, there was a significant effect of the stress induction on self-reported stress across groups, F (1,76) = 66.239, *p* < .001, Wilks’ *λ* = .541, *ƞ^2^* = .459. Post hoc test indicated that stress increased, *M_diff_* = 19.53, *SE*= 2.40, *p* < .001, *CI* [1.475, 2.430], from pre-induction (*M* = 23.3, *SE*= 2.39) to post-induction (*M* = 42.82, *SE* = 2.60, *p* < .001). There was no significant effect of ABM-condition, F (1,78) = .029, *p* = .866, *ƞ^2^* = .000, nor an interaction effect between Condition and Time points, F (1, 78) = .039, *p* = .844, *ƞ^2^* = .000, suggesting that conditions were not different in self-reported stress level during the induction.

Post-intervention, there was a significant effect of the stress induction on depressed mood across groups, F (1,76) = 52.596, *p* < .001, Wilks’ λ = .591, ƞ^2^ = .409. Post-hoc tests indicated that depressed mood increased, *M_diff_*= .418, SE = .078, *p* < .001, CI [.261, .574], from pre-induction (*M* = 0.69, *SE*= 0.091) to post-induction (*M* = 1.10, *SE* = 0.102, *p* < .001). On average, depressed mood increased by 59 % from pre- to post-induction. Twenty-seven percent of the sample reported decreased or no change in depressed mood in response to the stress induction post-intervention.

There were significant differences in depressed mood between conditions at each time point during the stress induction procedure, *F* (1,76) = 7.214, *p* = .009, *ƞ^2^* = .087, with the ABM group displaying less depressed mood at all time points compared to sham condition. However, there was no Time points x Condition interaction effect, *F* (1,76) = .006, *p* = .938, Wilks’ *λ* = 1.00, *ƞ^2^* = .000, indicating that the groups did not develop significantly different from one another in terms of depressed mood from baseline to post-induction.
